# A Manual of the Diseases of the Human Eye

**Published:** 1821-12-01

**Authors:** 


*A Manual of the Diseases of the Human Eye, intended for Surgeons commencing practice, from the best national and foreign Works, and in particular those of Professor Beer, with the Observations of the Editor, Dr. C. H. Weller: illustrated by four coloured Plates, representing thirty-seven diseased Eyes, and one Plate of Instruments: translated and illustrated by Cases and Observations, by* GEORGE C. MONTEATH, M. D. 8vo, 2 vol. Glasgow, Chapman, 1821.

A LATE ingenious writer,* who has taken a keen view of the progress which knowledge has made, and is likely to make, in this world, comes to the conclusion that, when society has fathomed all the depths of learning, and science found the boundary of its researches and experiments, the following will probably be *among* the most important changes —namely, that having learnt to be more careful of his health, man will be longer lived—that pleasures, simple and durable, will be more in request than vicious indulgence—and that "the *gastronomic* art will be esteemed the most important of sciences." We must confess, indeed, that the *stomach,* although, ex officio, the minister of the less rational part of man—the purveyor of the automatic life, or "*vie interieure,*" is, at the same time, a grand agent in the support of the moral man—of the soul itself, or (if the materialists will not allow us this particle of divinity) the sensorium commune. Small, we fear, would be the efflux of intellect from the brain, were it not for a corresponding influx of materials into the stomach. Homer, Horace, Burns, and Pitt, could vouch for the truth of this, were they on speaking terms with the present race. But while we grant

---

* Letter from Montagne the Younger, in N. Monthly Magazine, for October 1821.

a kind of physiological supremacy to the stomach, we must be convinced that there are other organs, the functions of which will continue to engross nearly as much of man's attention, to the end of time, as the "*gastronomic art.*" When will man cease to be charmed with the "*concord of sweet sounds*"—or fail to experience delight from a view of the beauteous and magnificent scenery scattered bountifully around him? The eye and the ear must for ever continue to be objects of man's most solicitous regard; and their diseases will doubtless attract a very considerable share of professional investigation to the remotest period of time.

Rapid as has been the progress of surgery in general during the last twenty years, the advance of the *ophthalmic* branch appears to have been the most remarkable of all—especially on the Continent, where it has been cultivated with enthusiasm. Priority necessarily secures a *temporary* superiority, in any art or science, and our German brethren look upon themselves as our masters in this department. At this moment, however, their superiority is very questionable, and there can be no doubt but that British genius, now that it is excited, will soon excel. Ophthalmic diseases and operations have now got into the hands of surgeons who have studied all the branches of surgery, and who are, consequently, best adapted for carrying this particular branch to perfection. It is not to *exclusive practice* that we object, provided it be preceded and attended by *general study*. There can be no more doubt that the regular surgeon, who devotes his time and attention to a particular train of operations, should be more *habile* than others in the performance of them, than that an able hospital surgeon should be more *au fait* with the knife, than he who rarely takes one in his hand. This observation applies more peculiarly to the ophthalmic operator than to any other. The eye of the well educated ophthalmic surgeon being more accustomed to contemplate the delicate textures of the visual orb, is more ready to detect the morbid processes going forward in them; while his hand, employed principally to poise and direct the finest and lightest instruments, acquires a delicacy of tact, and steadiness of motion, which cannot be expected, and would be frequently unnecessary, in that of the general surgeon, however dexterous in the most formidable operations. Let us not be misunderstood. We mean that in the capitals and larger cities of the world, where wealth can patronize and support the minutest division of labour, we see no reason why the regular surgeon, who studies his profession thoroughly, and devotes his attention principally to ophthalmic diseases and operations, should not restrict his practice, if so inclined, to that particular branch. At the same time, we are well convinced that the general surgeon, and even the general practitioner, may acquire, through the medium of those excellent public institutions now established throughout the world, such a knowledge of ocular diseases and operations as may qualify him for the discharge of this part of his duty, with honour to himself, and security to his patient.

The pages of this Journal have recently borne ample testimony to the excellence of British ophthalmology; but it is incumbent on us to lay before our readers an account of what has been done in those countries where the science has been cultivated still earlier, and with not less zeal, than among ourselves. Professor Beer's fame resounds from one end of the Continent to the other; and therefore Dr. Weller has obliged and benefited the profession by compiling, and Dr. Monteath by translating, the principal points of the professor's doctrines and practices in diseases of the eye. Without farther preface, then, we shall enter on our analytical labours, and lay before our readers a series of samples of what they are to find in the work itself, the momentum of whose circulation, though already great, we hope materially to accelerate.*

Dr. Weller's plan, in this "Manual," is very comprehensive; his arrangement is simple and perspicuous; his doctrines are comprized under the following heads—an introduction—a sketch of pure ophthalmia in general—of the diseases of parts which surround the eyeball—and of those of the eyeball itself.

Division I. This is altogether proëmial, and explains the differences between pure and specific inflammations of the eye. 1st. The *pure* completes its course without shewing any specific symptom either in the first or second stage, and hence the degree of inflammation only requires attention. It is distinct from idiopathic inflammation; for, although the latter be frequently, yet it is not always pure, because that which acted remotely on the eye, may also excite in that organ a specific disease already existing in the body. 2d. The *specific* originates from a peculiar quality of the living powers, which has already determined a general change in the vital functions. This kind assails particular textures of the eye, is distinguished by characteristic symptoms, and its treatment must be regulated by the degree of inflammation, the peculiarities of the affected part, the state of the constitution, and the nature of its determinative cause.

---

* The length of this article, and that of the succeeding Supplemental Review, obliges us to print the remainder of this number in small type. *Edit.*

Division II. *Pure Ophthalmia in general.* This, like every other inflammation, is characterized by topical redness, heat, swelling, and pain. Two distinct stages mark its progress. 1st. Together with the local symptoms, are frequent nictation and a flow of tears, which sometimes disappears, giving place to dryness and immobility of the eye-ball. In many cases, there is more or less aversion from light, and this sometimes ceases suddenly on the supervention of palsy. When the inflammation becomes intense, the general system is implicated. 2d. About the sixth day, a remission commences; the pain and heat abate; the arterial redness changes to a dirty-blue or brownish-red; effusions of blood sometimes occur. The eye is now more tolerant of light; the ocular secretions and excretions are renewed; in some instances the parts around the eye are affected with œdematous swelling; and, if the first stage was severe, or neglected, or arose from causes producing loss of substance, suppuration ensues.

Pure ophthalmia may be caused by impure atmosphere, excessive light, sudden transition from darkness to light, over-exerted vision, injuries and wounds, unnecessary employment of spectacles, improper application of eye-remedies, use of the kaleidoscope, friction of tartar-emetic ointment on parts too near the eye, encephalic and ocular congestion however induced, suppression of sanguineous discharges, dentition, and the irritation of carious teeth.

Particular circumstances will modify the treatment of either stage. 1st. All the exciting causes must be withdrawn, and foreign bodies of every kind removed. Dr. Monteath, in a foot-note, describes a very convenient method of discovering and extracting them, and illustrates its advantages by two practical histories. When severe spasm of the orbiculo-palpebral muscle is induced by mechanical or chemical irritation, the patient should be left in a dark room, and have a warm poultice of bread-crumbs boiled in water or milk, with some fluid opium, or leaves of henbane, applied over the parts. This being done, we institute an antiphlogistic treatment, general and topical, together with refrigerant draughts, tepid pediluvia, cooling aperients, and exclusion of light from the bed room. If much symptomatic fever prevails, abstraction of blood from the brachial, jugular, or angular vein, temporal arteriotomy, and local scarifications ought to be practised. An ophthalmia arising from suppression of menstrual, hæmorrhoidal, or nasal hæmorrhage, requires the application of leeches to the labia pudendi, around the anus, or on the alæ of the nose. Unguentum hydrargyri cinereum applied with a pencil will destroy crab-lice, which sometimes nestle in the conjunctiva of the eye-ball. 2d. When debility of the ocular vessels impends, they are to be invigorated by dropping vinous laudanum between the eye-lids, or by the use of astringent lotions and salves. The eye may now be exposed to a free, dry, warm air; and, if suppuration is established, to moderate light and the observation of agreeable objects. When the discharge is unhealthy, it will improve under the local use of tonic volatile remedies; and, if ulcers are formed, we bathe the eye-ball with tepid solutions of *lapis*

*divinus,** acetite of lead, sulphuric ether, or Peruvian balsam, containing a small proportion of vinous laudanum in each. Deep-seated ulcers require the lancet. In the suppurative fever, volatile stimulating medicines, with nourishing diet, are indicated.

Division III. *Diseases of Parts surrounding the Eye-Ball.* These are subdivided into two classes, 1st. Diseases of the external appendages of the eye—*blepharophthalmitis* inflammation of the eye-lids in general—*bleph. erysipelatosa,* erysipelatous inflammation of of the eye-lids—*blepharophthal. glandulosa,* inflammation of the glands of the eye-lids—*ophthalmia, neonatorum,* ophthalmia of new-born infants—*hordeolum,* stye—*anchylops erysipelatosa,* erysipelatous swelling of the eye's nasal angle—*tylosis,* callosity of the eye-lids—*chalazion,* hailstone—*scirrhus and cancer* of the eye-lids—*carbuncle* of the eye-lids—*œdema palpebrarum,* watery swelling of the eye-lids—*ptosis palpebræ superioris,* falling down of the upper eye-lid—*blepharosphasmus,* spasm of the eye-lid—*lagopthalmus,* hare's eye—*entropium,* inversion of the edges of the eye-lids—*trichiasis,* inversion of the eye-lashes—*ectropium,* eversions of the eye-lid—*anchyloblepharon et symblepharon,* adhesion of the edges of the eye-lids with one another and with the eye-ball—*pulpy, melicenous, and steatomatous, encysted tumours* of the eye-lids and contiguous parts—*vesicles,* millet seeds, warts, and mulberries of the eye-lid. 2d. Diseases of the organs situated between the orbit and eye-ball—*general inflammation* of the orbit—*wounds* of the orbit—*dacryoadenitis,* inflammation of the lachrymal gland—*scirrhus* of the lachrymal gland—*dacryorhysis,* flow of tears—*atresia,* or obliteration of the excreting ducts of the lachrymal gland—*dacryops,* encysted lachrymal swelling—*hydatis,* watery vesicle of the lachrymal gland—*inflammation* of the lachrymal sac—*hernia, dropsy, and fistula,* of the lachrymal sac—*encanthis inflammatoria,* inflammation of lachrymal caruncle—*encanthis schirrosa et carcinomatosa,* scirrhus and cancer of the lachrymal caruncle—*encysted swellings and aneurism* of the orbit.

We shall analyse the articles in this comprehensive list with all possible conciseness; to do more would dilate our sketch to a disproportionate extent.

*Blepharophthalmitis* commences in the edges of the eye-lid, which are tense and deep red; the swelling is very irritable and hot, and gradually extends to the verge of the orbit; the affected parts are exquisitely painful; there is pulsation in the eye-lid, with dryness of the eye-ball and nose; a sense of sharp-pointed bodies is

---

* The Lapis Divinus of St. Ives is thus prepared :—R. Nitratis potassæ, sulphatis cupri, sulphatis aluminæ, aa ℥viij. cortrita et mixta fluant in crucibulo, quibus sub finem addatur camphoræ tritæ ℥ss. rité agitata refrigerantur. It would have been an act of charity to common readers, if the translator had given intelligibility to the multitude of antiquated and outlandish epithets with which Dr. Weller's therapeutics abound.

felt under the eye-lid, and of dust in the nose occasioning sneezing, with pain in the eye and head; flashes of light pass before the sight; inflammatory fever arises as the disease proceeds; now begins the development of suppuration; the ocular secretions are restored; coldness is perceived in the eye; and an abscess gradually forms. Antiphlogistic treatment, general and local, is of course indicated under modifications determined by the degree of inflammation and the peculiarities of constitution. When there is abscess, it must be opened and treated according to the character it may subsequently assume.

*Blepharophthalmitis Erysipelatosa.* Its symptoms, causes, and treatment, are little dissimilar from those of erysipelatous inflammation of any other part. More active remedies, we believe, would be employed in such cases by our countrymen, than the continental oculists venture to recommend.

*Blepharophthalmitis Glandulosa.* Edges of the eye-lids affected with a bright-red, hardish, painful swelling; there is sometimes a sense of burning in the angles and edges of the eye-lids; the secretion of tears is increased; they are acrid, and redden or excoriate the cheeks; if the inflammation extends over the conjunctiva, the eye becomes dry, and the presence of granulary particles is felt under its lids; anon, the dryness, burning, and itching diminishes; viscid mucus exudes from the meibomian glands; the conjunctiva palpebrarum swells and is vascular; the angles and edges of the eye-lids are excoriated; the mucous secretion, by which the lids and lashes of the eye are agglutinated, more and more increases; it becomes puriform; and the disease has an exacerbation three or four hours after dinner or supper, when chylification has commenced. Early in the disease, it is to be treated with aqueous, or aqueo-acetic poultices applied cold and often renewed; when the muculent secretion has taken place, the eye is to be frequently bathed with weak solutions of the muriate of quicksilver in mucilage of quince seeds and vinous laudanum; when excoriations are present, they may be treated with Tanin's ointment,* in such strength as the parts in different subjects will bear; but if this produces no benefit, the lapis divinus may be tried; should the excoriations resist all these means and degenerate into indolent ulcers, the unguentum nitratis hydrargyri, or even the nitras argenti itself, may be cautiously employed.

*Ophthalmia Neonatorum.* A minute but not unfaithful account is given of the symptoms, causes, prognostics, and cure of this well-known disease; but we regard the treatment advised by Dr. Monteath, in a foot-note, as greatly preferable to the complicated exhibition of unguents, lotions, and salves, which our brethren on the Continent delight to employ. When the affection is recent, Dr. M.

---

* R. Adipis sui scrofæ ℥j. oxidi zinci impuri præcipitati, boli armenæ, aa ℥ss, hydrargyri præcipiti albi ʒii. M. fiat unguentum.

institutes an antiphlogistic course; after the second or third week, he practises scarifications of the eye-lids, even to the third or fourth time. He then applies blisters to the middle and back part of the scalp, and afterwards stimulates the abraded parts to give out a purulent secretion. So soon as the discharge and swelling of the eye begins to decline, he commences the use of astringents, and of these prefers a solution of the nitrate of silver, in the proportion of one grain and a half to an ounce of water. He applies it only twice a day, directs intermediate washings of the eye with tepid milk and water, and prescribes a daily exhibition of magnesia in doses regulated by the patient's circumstances and strength.

*Hordeolum.* An inflammatory circumscribed tumour, sited in a meibomian gland or the cellular substance on the edge of an eye-lid, is generally connected with scrofula or other cachectic maladies. When cold poultices and lotions fail in determining its resolution, it suppurates, opens, and emits puriform matter. Weller dresses the consequent ulcer with a plaster of semivitrified oxide of lead; we prefer occasional applications of the ammoniaret of copper or nitrate of silver, in the form of a mild solution. Some years ago, one of our patients, who had long been annoyed with successive attacks of hordeolous inflammation in both eyes, was effectually cured by an accidental ptyalism.

*Anchylops Erysipelatosa.* Here, the nasal angle bears all the characteristic marks of pure erysipelatous inflammation of the eye-lids; and, extending more or less to the neighbouring parts, runs a similar course, proceeds from like causes, and requires a particular modification of the same treatment.

*Wounds of the Ophthalmic and Palpebral Regions.* Their form and extent are determined by the agents with which they are inflicted: they refuse not, in general, to unite under the use of sutures or plasters and suitable bandaging. Amaurosis is induced by palpebral wounds in various ways:—by contusion, or extension, by partial or complete laceration of the superciliary nerve and its branches; by mere concussion or actual division of the retina; displacement of the other internal optic structures; and by concussion of the eye-ball and surrounding parts, accompanied with contusion and dispartition of the superciliary nerve. When they end in these or other diseased states, they require being treated according to the existing indications.

*Pacheablepharosis.* This is a knobby, hard, insensible, equable or uneven, swelling on the edges of the eye-lids. It occurs chiefly in scrofulous or gouty persons, and often causes the loss of the ciliary hairs. In addition to the proper constitutional treatment, narcotic or camphorated cataplasms, and mercurial salves, will complete a cure.

*Chalazion* exhibits a hardened stye which has resisted discussion or suppuration. Its cure consists in bringing on either of these states, by means of topical applications; when this course is ineffectual, excision will not fail.

*Palpebral Callosity and Cancer.* The marks of these states are not different in the eye-lids from what they assume in other organs: the cure depends on an early use of the knife.

*Palpebral Carbuncle* retains the ordinary carbunculous characters; it will yield to local scarifications and poultices containing decoctions of bark, with camphor and myrrh, and the internal use of cinchona and mineral acids.

*Œdema Palpebrarum* commonly appears in the upper eye-lid as a soft, unirritable, pale, doughy swelling, which friction, with warm stimulating preparations, can remove.

*Blepharoptosis* consists in a morbid relaxation and extension, with consequent puckering, of the integuments of the upper eye-lid, which cannot be raised. Cauterization, with sulphuric acid, the hot iron, and other means have been successfully used for its removal; but the best is excision of the cutaneous folds.

*Blepharospasmus.* An involuntary contraction of both eye-lids, forming almost always a symptom or sequence of other diseases; and, when these are expelled, it will retire.

*Lagophthalmos.* An unusual state, wherein the upper, sometimes the under, eye-lid appears shortened, and incapable of covering the ball. It is caused by relaxation of the affected eye-lid, or occasionally by spasm of its muscles, and also by ill-cicatrized wounds, accompanied with loss of substance; sometimes it is congenital. Dr. Monteath has frequently found it originating from a temporary palsy of one half of the face, produced by exposure to a current of air. From observations of several cases, he is convinced that cold is its invariable cause, determining at first an inflammatory state of the pes anserinus, and perhaps in some cases, an inflammatory swelling and diminution of the caliber of the aquæductus fallopii, causing pressure on the trunk of the nerve. Pain, for some time, radiates from the ear, along all the branches of the nerve; it then ceases, and perfect semifacial palsy follows. "This disease," he adds, "is soonest overcome by antiphlogistic treatment for the first few days, then by applying a semilunar-shaped blister around the ear, and rubbing the paralized parts with stimulating liniments." Dr. Weller combats the spasm with antispasmodics, the relaxation with topical stimuli and tonics, directing his attention, at the same time, to the general diseases from which these conditions arise. When the disease arises from cicatrices of the integuments, incision of the eye-lid and extension of the wound, till healed, have been tried, but without favourable results. Professor Dzondi continues frequently, for several weeks, to move the cicatrix backward and forward so as to dispart all its adhesions. He then divides it by a horizontal incision, loosening at the same time the subjacent cellular substance with the knife, to facilitate the stretching of the wound. The first dressing is dry, the rest with stimulating ointment, till granulation and cicatrization are completed.

*Entropium.* From the inversion of one or both lids, the eye-lashes irritate the ball, excite constant lachrymation, inflammation, and a panniform opacity of the corneal membrane. It is trouble-some, almost incurable, either by medicamental or chirurgical means. Saunders excided the tarsus as the best remedy, and seldom failed.

*Trichiasis.* Ciliary inversion constantly irritates the anterior surface of the eye-ball, producing inflammation, excessive lachrymation, with corneal pannus, specks, and ulcerations. When it accompanies inversion of the eye-lid, the entropic cure will also remove it; as a palliative, extraction of the inverted hairs may be practised.

*Ectropium.* The blear eye depends chiefly on eversion of the inferior eye-lid. When confirmed, it cannot be remedied; when it is sarcomatous, it may be treated locally with laudanum or sulphuric ether, with mercurial, antimonial, or escharotic salves, and by excision; when it arises from relaxation or palsy of the orbicular muscle, stimulating eye-waters will mitigate its effects.

*Anchyloblepharon* is, when adhesion of the palpebral edges with each other is, in various degrees, complete. When the eye-lid and surface of the optic conjunctiva unite to a greater or less extent, a *symblepharon* is formed, and, most frequently, has place in the upper eye-lid. Both forms of the disease are, though rarely, congenital; occasionally proceed from psorophthalmic excoriations, but oftenest from traumatic causes. Nothing but the scalpel can remove either of these states; and when the operation is indicated, it consists in severing the coherent parts by minute and cautious dissection.

*Pulpy, Melicerous, and Steatomatous* encysted tumours of the eye-lids and contiguous parts appear under peculiar forms, the nature of which may be ascertained by examination. Puncture of the tumour will dislodge its contents, and, if adhesive inflammation succeeds, a cure may be effected. Certain relief, however, can only be imparted by complete extirpation of the cyst.

*Watery Vesicles* of the eye-lids are to be opened, and subsequently washed with saturnine lotions, in which some laudanum has been mixed. *Fatty* vesicles, like millet grains, must be treated in the same manner, and the suety substance expressed. *Warts,* when they have a narrow pedicle, can be destroyed by ligature; when they are large, painful, and encircled by varicose vessels, are apt to degenerate into cancerous sores, and consequently should remain untouched. Violet-coloured moles on the outer surface of the eye-lid, and moriform growths on the inner, have been exterminated by applications of escharotic minerals.

*Inflammation of the Orbit.* Scrofulous, gouty, syphilitic people, are most susceptible of this rare disease. Obtuse, deep, increasing pain in the orbit, with immobility of the eye-ball and imperfect actions of the upper eye-lid, mark its commencement; forthwith, the eye-ball projects; vision is impaired; amaurotic blindness succeeds; there are manifest contraction of the pupil, complete unmoveableness of the iris, extreme sensibility of the eye-ball, and painful flashes of light; now the sclerotica, then the conjunctiva, reddens; the iris becomes arched and changes its blue or gray to a greenish, or its brown to a dark red; the pupil closes, the anterior chamber lessens, pain is exquisite, synocha prevails, the patient grows delirious, and the encephalic membranes inflame. At this period, the antecedent local symptoms acquire greater intensity; the sick person often shrinks and shivers; one or more red soft tumours arise between the eye-ball and orbit; they suppurate; œdema of the eye-lids supervenes; but, if one point of matter only appears, the eye-ball protrudes at that point, and the abscess does not implicate the rest of the orbit. Severe depletion, general and local, offers the only prospect of subduing the disease in its first stage; resolution being unattainable, suppuration must be promoted by the usual means; when fluctuation is quite evident, a large opening should be made with a lancet; then tents, moistened with laudanum and deposited in the ulcer, will induce granulation, and dispose the parts to cicatrize.

*Wounds of the Orbit.* Their general management is similar to that of other traumatic injuries. When incomplete prolapse of the orbit is caused by foreign bodies or infiltrations of blood, these must be withdrawn, and the organ replaced. If the eye-ball has been forced altogether out of the socket, and is itself severely wounded, if the optic chambers are full of blood, and if perfect blindness exists, then the whole eye-ball is to be removed.

*Dacryoademitis.* Its causes are obscure; its occurrence not frequent; its influence severe and dangerous; young people between seven and nineteen years of age, and whose constitution involves a scrofulous propensity, are exposed to it; generally it originates from the action of a current of air on the eye, and sudden changes of temperature. Progressive dryness of the eye, then an abrupt, fixed, oppressive, stinging pain in the temples, extending over the eye-ball, head, and face, are felt; the upper eye-lid, opposite the lachrymal gland, swells, becomes hard, dark-red, tense, glistening, exquisitely sensible: the conjunctiva reddens; the eye-ball feels hard and irritable; the lachrymal gland enlarges, pushes forward the eye-ball at the external angle, throwing the cornea towards the nose; vision declines; the pupil contracts; the iris becomes immovable; frequent flashes of light are perceived; mobility of the eye-ball is more and more limited; pain increases; synocha and delirium supervene; all the symptoms are aggravated; there is a troublesome feeling of coldness and weight in the eye; a suppurated point, in the conjunctiva of the eye-ball or external surface of the upper eye-lid, appears, and is accompanied with constant shivering. Powerful antiphlogistic means are required in the inflammatory stage; venesection, arteriotomy, leeching, cold poultices, spare diet, and free alvine evacuations, are indispensable; suppuration will be promoted by warm poultices, containing chamomile or hemlock leaves mixed with other emollient medicines; while deep hardness continues and the eye-ball projects, the swelling should be covered with a plaster combining hemlock and the semi-vitrified oxide of lead; opium will relieve great pain and restlessness; soon as fluctuation is distinct, the abscess must be opened; when the matter is discharged, a tent moistened with laudanum, almond oil, or any digestive ointment, should be placed in the bottom of the ulcer, to irritate the parts and induce the process of granulation; if the discharge becomes sanious, and a pale red bleeding fungus sprouts out, announcing a fistulous sore from caries of the subjacent bone, the opening requires to be dilated by the knife and tented with stimulating unguents, to promote exfoliation of the bone; should abscess of the lachrymal gland terminate in a fistulous ulcer, or if any callous capillary opening in the upper eye-lid remain, the nitrate of silver is to be carefully applied to the bottom of the sore. Our readers will readily discover, from the preceding and other accounts, that the continental ophthalmologists are absolutely ignorant of the decisive influence which calomel and opium, judiciously administered, exercise in re-balancing all inordinate actions of the nervous and vascular systems, on which the true inflammatory state indubitably depends.

*Scirrhus of the Lachrymal Gland.* It seldom occurs unconnected with a similar state of the neighbouring parts. Its chief symptoms are, the eye-ball pressed toward the nasal angle, also downward and outward from the orbit; it is dry, almost immovable, and cannot be turned toward the lachrymal gland; it is not painful and very little reddened; a firmly fixed, hard, uneven tumour appears in the temporal angle, and remains insensible till cancer supervenes; the eyelids are incrusted with half-dried mucus; the cornea is dim and even cadaverous. Beer rejects extirpation of the scirrhous lachrymal gland, and so will every honest surgeon.

*Dacryorhysis.* Unnatural secretion of tears varies, and requires suitable treatment. If it is a precursory or attendant symptom of pure ophthalmic inflammation, it ceases when the action of the primary cause is discontinued. When it remains after such a state, warm, dry compresses over the eye, with blisters, setons, astringent lotions and salves will tend to re-balance the functions of the part. If it is a result of scarlatinous, morbillous, variolous, syphilitic, or psoric cases, the topical treatment need not be different from that of the preceding kind; the general indications will be recognized in the character of the pre-existent disease.

*Obliteration of the Lachrymal Ducts.* From this arises a dry xerophthalmic and scheromatous state of the eye, which is immovable, and experiences a sensation of dust between the eye-lids. It is generally produced by traumatic causes, and can only be palliated by mild lotions with which the eye is to be often moistened.

*Dacryops.* This encysted lachrymal tumour enlarges when the eye weeps, and an elastic, insensible, circumscribed swelling, then appears in the superior eye-lid, near its temporal angle; pressing on this excites a sense of weight in the eye-ball and momentary flashes of light; when the upper eye-lid is raised and drawn out from the ball, and the tumour compressed, then the conjunctiva projects as a full roundish bag, in which the fluctuation of lymph is perceived. It is a very rare disease, remediable only by a nice operation, which our limits forbid our attempting to describe.

*Glandula Lachrymalis Hydatoidea.* This is a rare and formidable disease, whose causes are hitherto unascertained. It is thus described :—a cell of the cellular substance, connecting the individual glandules of the lachrymal gland, distend into a vesicle from the injection of tears which become acrid ; it is then separated from the membrane adhering to it, and forms an isolated hydatid lying among the lachrymal glandules; advancing in growth, it pushes forward the eye-ball from the orbit towards the nose, and, if its enlargement is slow, there is obtuse, deep pain in the orbit; the eye-ball feels as if extruded, especially on turning it round the lachrymal gland; slightly reddened the ball now projects from the orbit ; there is tensive pain in the eye and corresponding half of the head; dryness and difficult motion of the eye ensue; gleams of light flash before it; it becomes motionless; vision hitherto perfect is now double, inexact, or suspended; it grows confused and finally closes; the uninterrupted head-ache increases; the superior oblique muscle experiences incessant spasmodic movement: the eye acquires a dirty appearance, grows more like that of a dead person, and indicates approaching dissolution, even when the vital functions seem unimpaired; and, at last, a resisting hardness is distinctly felt in the temporal angle between the eye-ball and external edge of the socket. If its progress is rapid, the pain is more in the eye-ball itself, which, inflamed and enlarged, protrudes from the orbit, and, if not timely opened, suppurates, bursts under excruciating pains, and throws out a profusion of depraved matter and blood; but the organ does not yet collapse; it remains in the shape of an irregular fleshy mass external to the orbit; the head-ache continues; sleep and appetite are lost; the parotid of the affected side swells; and a resisting, firm, fluctuating hardness is felt in the region of the lachrymal gland. This disease, in its first stage, cannot be determinately known, nor the watery vesicle extirpated on account of the important parts lying in its vicinity; the following operation, therefore, is proposed as a palliative remedy. Under the upper eye-lid and near its external commissure, insert a small lancet, deep in the direction of the lachrymal gland, until the fluid issues from the vesicle, then introduce a piece of catgut or fine bougie, made of charpie and charged with saturnine water or salve, so as to form an opening through which the fluid can be discharged; through this the cyst should afterwards, if possible, be extracted with forceps; but if the eye-ball be already pushed out of the orbit, vision destroyed, and the eye of a dirty appearance, death-like, or severely inflamed, and consequently altered in texture, even this palliative practice will not succeed.

*Dacryocystitis.* This is oftener a sequela or concomitant of variolous, morbillous, scarlatinous, scrofulous, psoric, syphilitic, or arthritic inflammation, than a pure disease, and consequently its presence determines an appropriate modification of the treatment of each of these morbid states. When pure, it is harbingered by symptoms of a common catarrh; a bean-shaped, circumscribed, hard, sensible tumour forms in the region of the lachrymal sac, from which an obtuse pain extends to the eye-ball and nose; it reddens, and is intolerant of the slightest touch; tears trickle down the cheeks, and the nostrils are dry; erysipelatous inflammation seizes the neighbouring parts; general indisposition and fever exist. By this time, much mucus distends the sac; the nasal duct is obstructed; the anterior part of the sac swells and feels elastic even before suppuration has commenced; increasing secretion by the mucous membrane of the nose and lachrymal caruncle takes place; the disease's crisis impends; and, either the permeability of the nasal canal is restored and the mucous secretion improved, or the lachrymal sac suppurates, and, if unassisted, spontaneously bursts. Cold acidulated poultices over the parts, and frequent inhalation of cold water into the nostril, with leeching and general depletion, may be opposed to the primary symptoms; emollients will promote suppuration; the abscess ought to be laid open by a free incision with a lancet-shaped knife; the ulcer will granulate, obstruction of the ducts yield, and callous apertures of skin close, under an adequate exhibition of the ordinary means.

*Hernia of the Lachrymal Sac.* Distinguished by a bean-shaped, insensible swelling under the nasal angle, from which pressure removes a mucous fluid either through the lachrymal points or nasal ducts, and consequently empties the humour, but it soon fills again. Constant use of compresses moistened with astringentt fluids, and a leather cushion retained over them with a proper bandage, will ultimately reduce the extant sac.

*Dropsy of the Lachrymal Sac.* This is more elastic than the hernial tumour, and at last becomes discoloured. It is to be opened, the mucus squeezed out, the sac well syringed with warm water, and afterwards treated as a fistulous ulcer of the parts.

*Fistula Sacci Lachrymalis.* On the numerous methods of treating fistula of the lachrymal sac we decline at present to enter. We may add, however, that Dr. Monteath prefers Ware's styles to catgut in the treatment of fistula lachrymalis, and either of these to the tedious use of injections, salves, and internal remedies. In three cases, with a large scrofulous fungus sprouting from the aperture in the sac, he has seen the excrescence disappear, and a perfect cure effected by the mere wearing of the style. For hernia of the lachrymal sac he asks, "Is there reason to think that a free incision of the sac, even cutting across the round tendon of the orbicularis palpebrarum, and tenting the wound with escharotic dressings until the sac contracts by inflammation to a natural size, and dilating the nasal canal at the same time, if necessary, would be attended with success?"

*Encanthis Inflammatoria.* Here the lachrymal caruncle and semi-lunar valve swell, become vascular, and painful; absorption of tears into the sac ceases; the lachrymal points and whole conjunctival membrane inflame; if suppuration ensues and is neglected, excrescences rise from the ulcerous surface, and often acquire a large size. Attempt resolution by the usual means; apply desiccative remedies to the ulcer, and escharotic ones to the morbid sprouts.

*Encysted Tumours of the Orbit.* Real encysted tumours containing a compact, fatty, or puriform mass, occasionally though rarely occur in the cellular substance which surrounds the eye-ball. Their seat in the orbit is sometimes deep, sometimes superficial, generally under the eye-ball, seldom at its side. As the tumour enlarges, it presses the eye-ball so much outwards and upwards, that it acquires a peculiar appearance. Tears run constantly over the cheek; there is aversion from light; and, during the return of inflammation in the eye-ball, the local pain extends over the whole head. This infrequent disease can only be cured by extirpation, which is done in the following manner:—The under eye-lid being made tense, the skin and orbicular muscle, in the direction of its fibres, are to be divided by a bistoury with a convex edge; the sac must then be seized with a suitable hook, drawn out from the orbit, and carefully extirpated. A bandage and graduated compresses, by moderate pressure, will replace the eye-ball. Dr. Monteath excided two of these tumours. In one case the eye-ball also was removed. "The tumours," he says, "exceeded the size of the eye-ball, lay directly behind it, and so completely encircled the optic nerve, that the latter was diminished one half in thickness by the pressure of the tumour. Vision had been rapidly declining previously to the operation. This tumour was exceedingly hard, of anomalous texture, and surrounded by a layer of condensed cellular substance. Its anterior aspect touched and pressed upon the posterior aspect of the eye-ball, but had no connexion with it, except through the medium of the optic nerve and cellular substance." The patient, a young woman, continues in perfect health, after the lapse of more than two years since the operation.

*Orbital Aneurism.* Few cases of this description have been observed; they are always preceded by ocular and encephalic pains; pulsation in the eye begins and increases; on touching it, a sense of rushing and thrilling is felt; head-ache becomes more frequent; humming in the ears takes place; and the eye is pushed sometimes out of the orbit. Obliterate the carotid of the affected side by ligature, or leave the patient to his destiny.

Division IV. *Diseases of the Eye-Ball, preceded by Observations on Wounds of that Organ.* This is subdivided into four classes. 1st Diseases of the transparent parts of the eye-ball—*conjunctivitis*, conjunctival inflammation—*pannus*, thick film of the eye—*pterygium*, winged film—*pinguecula*, fatty film—*fleshy* and *fatty fungus*, and *papula*, of the conjunctival membrane—*keratitis*, inflammation of the cornea—*opacities, specks, and conical projections* of the cornea—*keratokelé*, hernia of the cornea—*lentitis*, inflammation of the lens—*cataract glaucoma*, glaucomatous opacity of the vitreous humour—*synchysis*, solution of the vitreous humour—*hydrophthalmia*, dropsy of the eye—*iritis*, inflammation of the iris—*mydriasis, myosis, et atresia pupillæ*, dilatation and immobility, unnatural contraction, and obliteration of the pupil—*artificial* pupils—*retinitis*, inflammation of the retina—*amaurosis*, nervous blindness—*strabismus*, squinting—*luscitas*, oblique vision—*myopia*, short-sightedness—*presbyopia*, far-sightedness—*diplopia*, double vision—*nyctalopia*, day blindness—*hemeralopia*, night blindness—*fungus hæmatodes* of retina—*ophthalmitis externa et interna*, external and internal inflammation of the eye-ball—*staphyloma* of the cornea and of the iris—*synechia anterior et posterior*, adhesion of the anterior surface of the iris with the cornea, and of its posterior surface to the capsule of the lens—*oph. universalis*, inflammation of the whole eye-ball—*atrophia bulbi*, wasting of the eye-ball—*cirsophthalmia*, general varicosity of the eye—*scirrhus, cancer*, and *ossification* of the eye-ball—*congenital* deficiency and superfluity of eyes—*specific inflammations*, including the catarrhal, rheumatic, gouty, variolous, morbillous, scarlatinous, syphilitic, gonorrhœal, iritico-syphilitic, syphilitico-scorbutico-blennorrhœal, psoric, scrofulous, dacryoblennorrhœal, and scorbutic ophthalmics.

Prosecutively of the method we had assigned ourselves, we again go on and submit an analytical view of the many important subjects of which this part of the manual professes to treat.

*Wounds of the Eye-ball.* Circumstances, such as may be appreciated by any "commencing" oculist, will direct the treatment of these. Their mechanical or chemical origin distinguishes their classifications; their individual symptoms are various but determinable. A singular instance of disruptured eye came under Dr. Monteath's observation. The patient, in attempting to separate two men who were fighting, received a blow on one of his eyes; the ball was burst, and vision instantly destroyed. On another occasion, he was standing near a man and woman who were quarrelling. The woman, in a violent passion, turned round suddenly, and unintentionally struck the man's sound eye with her elbow. This eye shared the the same fate, and its vision was ruined. Both eyes burst one line from the cornea, on the nasal aspect of the eye-ball. When examined, the pupils were distorted and drawn towards the rupture, and a capsular cataract existed in both eyes. The retina being insensible, no operation was advised.

*Conjunctivitis.* This is distinguished by the marks of inflammation, as it appears in all the optic membranes, and, in each of its stages, demands a similar management.

*Stannus* consists in the conjunctiva of the sclerotica being interwoven with small and red blood-vessels, between which a grey whitish-red dimness is observed; when it is considerable, its vascular net-work appears so thick that the limits between the sclerotica and cornea can hardly be distinguished, while the iris and pupil are still more indistinctly seen. It frequently originates from the irritation which trichiasis or ectropium produces. Often, however, it is a symptomatic disease. Leeches and emollient poultices will check its inflammatory stage; if it is ancient, or produced by cachectic affections, laudanum, with æther or mild escharotic powders, should be introduced into the eye; when these fail, issues, blisters, scarifications, and partial excision of the largest vessels may be advantageously employed. In obstinate cases, Dr. Monteath cuts out a portion of the conjunctiva, from a line to a line and a half in breadth, all round the edge of the cornea. On the second day after the operation nearly two lines of the corneal verge will have become transparent, and the clearing goes on progressively from the circumference to the centre. Owing to the density of its tissues, the latter part may not regain its transparency for weeks or months. When the pannus is scrofulous, attention should be directed to the improvement of the patient's general health, and his eye left undisturbed.

*Pterygium* proceeds from a triangular thickened fold of the conjunctiva, for the most part originating from the nasal angle, and connected with the semilunar membrane. It is either thin, half transparent, or greyish, and furnished with few blood-vessels; or thick, filmy, flesh-like, cartilaginous, and vascular. When thin and recent, it will vanish under the external use of powdered sugar and alum, or similar applications; when thick, it is most safely and speedily cured by the knife.

*Pinguecula* is a small, flat, dirty yellow film, lying under the conjunctiva and near the temporal angle. It occasions deformity, but seldom interferes with vision. If its removal is desired, scarifications, or, preferably, the scalpel will execute the task.

*Fleshy and Fatty Fungus.* In scrofulous or syphilitic persons, fleshy excrescences arise on the limits between the cornea and sclerotica, and produce hairs of considerable length. Excide them, and apply astringent or mild escharotic remedies to the part.

*Conjunctival Papula* is a roundish, hard, pale-red, itchy swelling, not larger than a pin's head, and sited in the conjunctiva of the inferior eye-lid, near the semilunar fold and lower lachrymal point. It is commonly dependent on irregular or defective menstruation, and disappears as that is remedied. If it does not disappear, seize it with a hook and cut it out.

*Keratitis.* Inflammation attacks the cornea, which becomes dim, muddy, and reddish, from slight enlargement of its vessels. It yields to antiphlogistic remedies, topically and generally employed.

*Corneal Opacities and Specks.* These exhibit a great diversity of shapes; but, with the exception of the bony kind, their nature is nearly uniform. Local means generally dissipate them, and these are either emollient, resolvent, stimulant, or escharotic drugs, and division of the enlarged arteries by which they are fed.

*Conical Projection of the Cornea* arises from causes very little known; among others, from severe crying in difficult parturition. The young are generally, the old occasionally, attacked by it; sometimes it exists in one only, sometimes in both eyes at the same time. It consists in a pyramidal protuberance of the corneal membrane, advancing slowly and without inflammation. Its apex thickens, sometimes becomes opaque, and always forms the central point of the tissue which is its seat. When viewed laterally, it resembles a hard crystal; and, by preventing the proper view of the iris and pupils, it produces short-sightedness, and such a disordered refraction of the rays of light that even very near objects seem confused and imperfect. For its cure, the aqueous humour has been evacuated, and subsequent compression employed, but without benefit; infusion of tobacco dropped, several times a day, into the eye, is more beneficial; depression of the lens is sometimes of advantage, but is uncertain, and, in many cases, inapplicable. Although it seems to be nothing else than a dropsy of the aqueous humour, yet it is certain that medicines directed against such a dropsy are altogether inefficacious in removing this affection.

*Keratocelé.* Corneal hernia is a projection of the internal tissue of the membrane, in forms of a greyish, semi-transparent, watery vesicle. Fluid astringent and escharotic medicaments dropped into the eye promote its disappearance and prevent its return.

*Lentitis.* Lentic inflammation is a state generally precursive of cataract. Aversion from light, nebulous dimness of vision, and increased vascularity of the pupil, announce its development; a fine wreath of vessels in the anterior capsule runs concentric with the round pupil; this zone consists of several vascular arches, to which many others, from the periphry of the capsule or iris, extend in a radiated manner; a similar zone is often observed in the lens itself; between the blood-vessels of the capsule and lens is found effused coagulated lymph, by which vision is still more obscured; slight pain only is experienced in the bottom of the orbit, and infra-orbitary region. This symptomatological sketch induces us to the pathology of cataract, of which Dr. Weller's account is practical and minute.

*Cataract.* This inexpressive epithet is applied to every dimness of the lens, or of its capsule, or of both structures at the same time, by which the rays of light are more or less interrupted in their passage to the retinal expansion. Commencing cataract is often confounded with incipient amaurotic blindness; their symptoms may be compared. 1st. In the former, the iris retains its mobility, but objects appear enveloped in a cloud, dirty and dusty; decrease of sight exists in proportions corresponsive to the dimness visible behind the pupil, in the centre, seldom in the edge, of which it is first seen; as cataract advances, the pupilary verge is surrounded by a blackish ring, which is the iridic shadow upon the lens, now rendered visible from its opacity; when cataract begins in the middle of the lens, it obscures objects presented immediately in front of the eye; the patient, however, knows whatever is placed to one side, and sees better in the shade than in bright day; for some time the flame of a candle seems enveloped in a whitish cloud; but as the disease advances, even this imperfect vision wanes, and the glare around the flame can only be perceived. 2d. When amaurotic blindness is impendent, the opacity has a deeper seat in the eye than the lens, and appears concave; its colour inclines more to greenish or reddish than to grey, though the dimness be inconsiderable, the patient is often almost blind; the pupil is dilated, and its edge more or less angular; the iris slightly or not at all moveable; the cornea dull; when the disease retains a paralytic nature, the eye loses its brightness, and the contour of a flaming light appears coloured like the rainbow. Alternate increase and diminution of sight depend not, in such a case, upon the pupilary dilatations and contractions, but on what causes invigorate or debilitate the whole frame.

*Lenticular Cataract.* This species lies considerably distant from the uvea, and exhibits no cloudy, bright-white specks; it begins in the centre of the lens with a yellowish grey colour, shaded off towards the edge; at the same time, the shadow of the pupil's margin represents a blackish ring, but that aperture itself ceases not to dilate and contract.

*Capsular Cataract* commences in the pupil's edge, in the indefinite form of white points, stripes, or specks; its colour is very bright, but unequal; it generally terminates in the capsulo-lenticular kind. Three varieties of it have been defined. 1st. *Anterior.* when the anterior half only of the capsule is opaque, and contains bright-grey, chalky-white, or leucomatous specks and stripes, it becomes thickened, sometimes fills the posterior chamber of the eye, consequently impedes the mobility of the iris, and intercepts the iridian shadow; and vision is deeply injured, nearly annihilated. 2d. *Posterior*—wherein the posterior half only of the lentic capsule is opaque, with muddiness, concave, greyish-white, irregular, but no chalky-white specks and stripes, uninterrupted mobility of the iris, and less impairment of sight. 3d. *Complete*—distinguished from the first variety by the posterior chamber of the eye being entirely destroyed by thickening of the capsule, the pupil being almost immoveable, and the iris sometimes arched.

*Choroid Cataract,* characterized by brownish, arborescent figures, on the anterior capsule of the lens, which may either become opaque, or continue transparent. Its nature is little known. Some ancient oculists regarded its brownish branchings as prolongations of the choroid coat. Beer considers them as the impression of the uveal tapetum upon the capsule of the lens; others believe they are in the substance of the capsule itself, or imagine that, being inflamed, its blood-vessels have been described under this name.

*Morgagnian Cataract* consists in a muddiness of the morgagnian humour, and is produced by sudden, chiefly chemical, influences upon the eye. The lens changes into a milk-like fluid, and perfect capsular, or capsulo-lenticular cataract supervenes. Its colour is milky, soft, and delicate, and the whole pupil seems cloudy; but the clouds change their form every time the eye-ball is rubbed, or quickly or smartly moved; the posterior chamber is filled up, and vision more or less obscured.

*Capsulo-lenticular Cataract.* Here the opacity, when close to the iris, is partly chalky-white, partly like mother-of-pearl, and, in many parts, these shades are stratified upon each other, in such a way that the pearl-coloured always lies above the chalky-white layer; the iris is immoveable, but the pupil continues round; and, from the cataract's pressing the iris towards the cornea, the patient's perception of light is very imperfect. Its varieties are six. 1st. That which is accompanied with formation of new substance in the anterior capsule. It is *marmoraceous*, *fenestrated*, *stellated*, *central*, *punctated*, *or dimidiated*, according to the different forms which the new structure assumes. In almost all the varieties of the capsulo-lenticular cataract, the substance of the lens, with exception of its nucleus, becomes milk-white, or gelatinous. 2d. Encysted, which lies at different distances from the iris, according to the situation of the patient's head, and is distinguished by its snow-white colour; it often appears tremulous or swimming, and the cause of this depends on the connexion of the lentic capsule with the neighbouring parts having been weakened or destroyed. 3d. Pyramidal, originates always from violent inflammation of the eye-ball, and is characterized by a white, almost glistening, cone-shaped growth, projecting through the pupil, and rising from the centre of the anterior capsule of the lens. Its adhesion to the pupil's edge renders that aperture angular, the iris immoveable, and the sight weak, or entirely wanting. 4th. *Dry-hulled*, consists in a drying of the lentic nucleus, and shriveling of the capsule. It occurs to very young children, in whom frequent convulsions, particularly of the ocular muscles, have partially loosened the capsule of the lens from its connexions. Its colour is bright grey-white; its size small; its distance from the iris considerable. The motions of the iris in this kind are generally free, and the sight never entirely destroyed. In adults, it is always of a dazzling white, with a few dim specks inclining to yellow; it is also flattened; and a mere sense of light alone remains. 5th. Bursal, known by a bag of fetid, ichorous matter between the lens and the posterior part of its capsule; by a duskish, orange colour, sluggish motion of the iris; want of the posterior chamber, slight arching forward of the iris, very indistinct perception of light, and by an evidently debilitated and cachectic habit of constitution. 6th. *Trabecular*, marked by an anterior capsulo-lenticular cataract formed behind the pupil, which is contracted and angular. To the cataract is attached a chalky-white, glistening, thickish bar, having a perpendicular or horizontal direction, adhering by both ends to the pupilary edge of the uvea, and rendering the iris motionless, the eye-ball atrophic, and vision indistinct, or annihilated.

Such are the species and varieties of cataract; its other differences depend upon the consistence of the lens and capsule. 1st. *Hard*, occurs mostly in old people, and is always lenticular; it is darkish or black, and reddish when held opposite light; vision is only limited, not lost; the motions of the iris are vigorous. 2d. *Firm-tough* cataract chiefly belongs to the capsular and capsulo-lenticular kinds. 3d. *Cheesy*, or gelatinous, has its whole substance or only its surface *soft*. It is always of a bright-grey, or greyish-white, or sea-green colour, unequally distributed. In this, the sight is often destroyed, and the iris's motions sluggish. 4th. *Fluid* is very difficult to be discovered, by reason of the capsular opacity which accompanies it; vision, however, is not lost. When this opacity is only partial, the cataract, on bending the head forward, lies upon the uvea; but, if the head is held quiet for some time, a thicker sediment above, and a thinner structure below, are observed. 5th. *Fluid-hard*, is exemplified in the morgagnian and bursal cataracts. Besides all these, they may also be distinguished into the mature and immature, or the purely topical, and the locally, constitutionally, and perfectly complicated.

Cataract is often hereditary or congenital, and may originate from different causes—from ossification and closure of the lentic and capsular vessels at any period of life, chiefly in old age; from long misuse of the eyes; unusual, sudden, and strong light; protracted insolation of the head and eyes; working over bright fires; local effects of concentrated mineral acids and æthers; spirituous liquors taken to excess; ophthalmic and lenticular wounds; and, in most instances, from inflammation of the transparent ocular structures.

*Perfect* cataracts cannot be cured by medicines; incipient ones have yielded to mercurials, antimonials, digitalis, belladonna, and pulsatilla, in powder or extract. An eye-water of henbane and opium will dissipate muddiness formed by existing lentic inflammation. But the operation alone offers the best prospect of a cure; and, for it, the patient should be prepared by venesection, alvine depletion, temperance, and mental tranquillity.

Cataract may be removed—by depression—by depression and reclination—by extraction—and by keratonixis, cutting it into little pieces with a needle introduced through the cornea. Each of these processes, and the subsequent treatment, are described with comprehensiveness and precision; but the length to which this article must necessarily extend, precludes our attempting to transfer Dr. Weller's operative precepts into the pages of this Journal.

*Secondary* cataract is either lenticular or capsular, and occasionally succeeds the operation. The first is induced by remnants of the lens rendering the pupil opaque. Bags of aromatic herbs may promote its absorption; but, if this do not take place, the smaller particles are to be depressed, the larger reclined, and if a perfect cataract remains, it must, if possible, be extracted. The other exists when vision is obscured by the lentic capsule. When the opacity is considerable, the capsule requires to be torn in all directions by the needle, and its flakes removed from the pupil.

We shall now conclude this branch of the subject with a case of *boney cataract*, for which the translator himself operated with complete success. A shoemaker, sixteen years prior to his consulting Dr. Monteath, sustained a violent stroke on his right eye. Ten years after this, a surgeon attempted to couch the cataract, but failed. In January 1818, this man was attacked with acute pain in the eye and head, which did not yield till after three months of severe medical and surgical treatment. In November following, the same pain recurred with violence. His sufferings now became agonizing the moment he raised his head from the pillow, deprived him of all comfort, and rendered him incapable of working. His surgeon in vain tried every possible means for his relief. He was leeched to a great extent, scarified often, freely bled from the temporal artery, repeatedly blistered over the head, underwent a three months' course of mercury. After ten months of dreadful suffering, debilitated and despondent, he came under Dr. M's care. The eye was now excessively tender, and considerably inflamed, the pupil much dilated, and filled with a yellowish, chalk-coloured projecting cataract, which nearly occupied all the anterior chamber. This cataract was firmly embraced by the iris, into whose aperture it had by some cause been forced. Extraction of the lens was advised; from a large incision of the cornea the cataract was removed with a hook. It was a perfect shell of bone, of the size and shape of the lens, and complete on all sides, except where the central artery enters the posterior capsule. The eye was dressed as usual, and the patient felt instant relief from all his sufferings. In a few days the corneal wound had adhered, and soon healed. On examining the eye, when free from all tenderness, it was found to have resumed its natural form and size.

*Glaucoma.* It always evinces itself as an arthritic iritis, by increasing, piercing, and rending pains, bursting as it were the eyeball, during which, along with muscæ volitantes, the pupil dilates and becomes elongated towards both angles, and the sight progressively wanes. As opacity of the vitreous humour advances, the lens grows cataractous, assumes a greenish-grey aspect, increases in circumference, fills the posterior chamber, pushes the iris forwards, seats itself in the enlarged pupil, diminishes the anterior chamber. The eye-ball now decreases, and becomes atrophic; the eye-lids fall in, and shut for ever. Medicine here will altogether fail; if any treatment be instituted, let it not be different from that of irritic inflammation sequencial to repressed gout.

*Synchysis* arises as a sequence of syphilitic iritis, or of the over-use of mercury. In it the vitreous humour loses its transparency, becomes muddy, and brownish-red; vision is weak or destroyed; or, if retained in some degree, the patient is far-sighted, his pupil contracted, and more or less angular; the iris does not expand and contract, but undulates during violent motion of the eye; the vitreous humour becomes thin as the aqueous; its cells are gone; and the hyaloid membrane tears from the slightest causes; the lens is cataractous, soft, white, and cheesy; the eye-ball is also soft; the sclerotica falls into folds; and atrophy of the eye finally supervenes. Complete synchysis is incurable.

*Hydrophthalmia* exists in three diversities of form—from too great quantity of the aqueous humour—from morbid increase of the vitreous humour—and from a complication of both these states.

1st. In this, the cornea's circumference increases to four or five times its diameter, without bursting; it still remains transparent, but at a later period becomes muddy; the anterior chamber enlarges; the iris ceases to move and grows darker; the pupil is neither contracted nor enlarged; weight, with tension and oppression, is felt in the whole eye-ball; at the outset, there is far-sightedness, latterly amaurotic amblyopy, and difficult movement of the eye; and the sclerotica, around the corneal margin, obtains a bluish cast. Its causes can seldom be discovered; its cure is uncertain. When it has been consecutive to a general disease, that disease must be first cured; if it is local or the effect of ocular inflammation, give digitalis with calomel, aqueous drinks containing supertartrite of potash with borate of soda, and apply blisters, setons, and similar means; when it arises from repelled eruptions, reproduce these or artificial ones, and exhibit internally camphor, sulphur, and the precipitated sulphuret of antimony. At the commencement, dry, warm bags of aromatic herbs should be placed over the eye, and the supra-orbitary region perfricated with mercurial and stimulating unguents, or liniments; when such means fail, as they generally do, the cornea, when the eye is not varicose, must be punctured, and the aqueous humour evacuated.

2d. Dropsy of the vitreous humour shews the posterior half of the bulb enlarged, and the cornea projecting in a conical form, without increasing in circumference or losing its transparency; the iris is motionless, and arched forward; its colour is unchanged; the anterior chamber sometimes obliterated; the pupil is partially contracted, dilates when the untorn vitreous humour presses forward the lens; the sclerotica is distended, its corneal margin bluish and impure. Early in the disease, the eye is short-sighted, afterwards weak-sighted, ultimately blind. In this kind, the motion of the eye-ball and eye-lid is soonest impeded, pain in the eye and affected side of the head, want of sleep, and loss of appetite, progressively increase, and often drive the sufferer almost to despair. At last the cornea bursts, the fluid escapes, the eye-ball sinks and becomes atrophic; or, in cachectic subjects, the ball degenerates into a fungous mass, grows indurated, and proceeds, with excruciating pains, to form a cancerous ulcer. Its causes remain indefinite: not seldom it follows gout, syphilis, scrofula, or ophthalmic inflammation; it may yield to some active modification of the treatment prescribed for the first variety, but failure in this is more certainly inevitable.

3. When both kinds combine, there is a very varicose state of the eye-ball, which frequently attains an enormous size, projects from the orbit, and exhibits the true buphthalmic eye. Here local means must never be used; a palliative and constitutional treatment alone is indicated; when this is unprofitable, extirpation of the morbid organ waits in reserve. Even this sometimes ends in the supervention of a spongy carcinomatous fungus in the orbit, which gradually extinguishes life. In an elaborate foot-note, Dr. Monteath introduces a case of hydrophthalmia combined with a tremulous capsulo-lenticular cataract, which occurred in his own practice. Our readers, at Vol. II, p. 142, 451, of the Medico-Chirurgical Journal and Review, for 1819-20, will find the detail of some cases of ophthalmic dropsy treated on principles drawn from a particular view of the disease, nature, and development.

*Iritis* sets out with obtuse, heavy, deep pain in the eye, dimness of vision, pupilary muddiness, increasing aversion from light, progressive diminution of all the iridial motions, without affecting the pupil's circular form and position, or its contractions. At first, the small iridian circle, afterwards the large one, assumes a deepening colour, the floating curtain, when grey or blue, becomes greenish; and reddish, when brown or black; it also swells and projects; vision fails, and pain increases and spreads as the inflammation extends over the anterior capsule and deeper structures of the eye; there is synochal fever; the sclerotica is rose-red, with the redness shading off toward the circumference; and the cornea loses its peculiar lustre. These symptoms attend the first stage; as the second advances, the pain becomes fitful; there are flashes of light; augmented conjunctival redness; angularity of the pupil, which contains a delicate albuminous mesh, determining cohesion of the iridian circles with the inflamed anterior capsule of the lens; lymphatic exudation into the pupil now supervenes, and sight is destroyed; projection of the iris goes on; the cornea waxes dimmer; small, yellowish-red, round elevations in the iris arise; there are abscesses which burst and form real hypopious ulcers. If the iritis implicates the retina, vitreous humour, and choroid membrane, vision is irreparably lost; if it invades the external optic tissues, the iris gradually protrudes, sometimes unites with the cornea, and induces a staphylomatous state of that membrane.

Recent iritis requires such modifications of the antiphlogistic treatment as are generally applicable to internal ophthalmic inflammation. When vision is destroyed, and a corneal staphyloma impends, the eye may be stimulated by injections of laudanum and sulphuric ether. On the excitement being moderated, tepid solutions of the extracts of henbane or night-shade will impel the radiated fibres of the iris to contract, and the pupil to dilate, if the remedy overcomes the contraction of its circular fibres. When coagulated albumen is deposited in the pupil, the action of night-shade may be assisted by remedies which promote absorption. Among others, opiated mercurial lotions, liniments, and ointments, with calomel internally, may be tried.

*Pupilary Anomalies.* *Mydriasis* may be an effect of encephalic or ophthalmic dropsy; of vermiginous, amaurotic, hysterical, or hypochondriacal disease; or of genuine iridial palsy. Adhesions of the uvea, with the anterior lentic capsule, and protracted exposure to darkness, will induce it: it is sometimes congenital. Attempt its cure by removing the primary disease, blistering the eye-brows, and by the internal and external use of antiparalytic medicines. Palliate it by shading the eyes, and using tubulated spectacles. *Myosis* is sometimes congenital, sometimes symptomatic of hypochondriacal, hysterical, and other nervous affections. Observation of minute, glittering objects, determines it; occasionally it is the offspring of internal ocular inflammation. Remove the morbid state from which it proceeds, confine the patient to a dark chamber, advise a green shade, tubulated spectacles, or the introduction of belladonnian extract into the eye, as means of cure. *Atresia Pupillæ* is rare; it is often congenital; it follows inflammation of the eye, absorption of matter, extravasated blood, and adhesion of the iridian and corneal surfaces. The congenital is generally removed by spontaneous disruption; otherwise, the operation for artificial pupil is required.

*Artificial Pupils* are formed in three ways—by *corotomy*, incision of the iris; *corectomy*, excision of a part of that membrane; and *corodialysis*, tearing the iris from its ciliary connexion.

1st. Incision of the iris, discovered by Cheselden, and improved by Janin, is now little used. M. Maunoir has invented a different method by which it may be performed. He introduces into the anterior chamber, through a previous incision of the cornea, his corotomic scissors; gently opening them, he perforates the centre of the iris with the lower sharp and pointed blade, pushes it along the posterior surface of this membrane, till the upper blade, which is furnished with a knob, has reached that part of the anterior chamber where the cornea is united to the sclerotica, and cuts at once through the iris. A second incision, diverging from the first, is now made in the same manner and in such a way that the cuts form a V, the point of which is in the centre of the floating curtain, whose circular as well as radiated fibres soon act, and an orthogonal or semilunar pupil restores vision. Another method, a simpler one indeed, has more recently been projected by Sir William Adams; but, lest our feeble voice may not be heard amid his iotacismal pretensions to originality and perfection, we resign to the ophthalmological knight himself the innocent pleasure of vociferating his own excellence—of promulgating his own praise.

2. Excision was first performed by Wenzel the father; from Gibson it received some modifications; Beer performs it in the following manner. Having, with the cataract-knife, divided the cornea one line in length, and as near the sclerotica as possible, if the iris be no where attached to the cornea, and extrudes through the incision, he seizes the projecting part with a hook, and clips it off in a line horizontal to the lips of the wounds. This done, the iris soon retracts, and a well-shaped pupil appears. When the iris adheres to the cornea, and the pupil is only distorted, on finishing the corneal incision, he introduces the hook through the wound, so as its point is neither directed toward the iris nor the cornea, seizes with it the pupilary edge of the iris in an oblique direction, draws it through the opening, and clips away the protracted part. If the pupilary edge of the iris adhere to the cornea at the spot, where the artificial pupil should be made, the larger iridial circle must be seized by a hook or forceps, and the necessary portion excided. When a central leucomatous opacity intercepts the passage of the rays of light through a regular pupil, Himly opens the cornea near its periphery, introduces a hook into the anterior chamber, lays hold of the iris's pupilary margin, draws it through the incision, and leaves it fixed in the wound, when it unites with the cornea, and the oblique pupil appears at the leucoma's side. If the cornea and iris are destroyed, Autenreith forms a pupil, by cutting out a part of the sclerotica and subjacent opaque tissues; and, after this, the wound gets covered with a thin, pelloroid film, through which vision may be possible.

3. Corodialysis was originally practised by Schmidt, Buzzi, Assalini, and Scarpa; Reisenger, Langenbeck, Himley, Graëfe, Wagner, and others suggested improvements of the process. Reisenger makes a small opening in the cornea, introduces hooked forceps, through the anterior chamber, as far as the iridian circle; turns their points toward the iris; opens the forceps; catches and hooks the iris; separates it from the ciliary ligament; recloses the instrument; draws part of the iris through the corneal aperture; and entangles it there, where adhesion secures it from retracting.

*Retinitis.* Bright light, looking long and steadily at glistening objects, suppressed nasal hæmorrhage, and every thing capable of irritating the retinal expansion, originate this disease. It is harbingered by aversion from light, lachrymation, head-ache, and deep stinging pains in the eye; then, glances and sparks of fire darting before the eye-ball are perceived; and sometimes a complication of the symptoms of choroideal, iritic and sclerotic inflammation is superinduced. By the remedies which allay internal ophthalmitic excitement the retinitic will in general be subdued. Should an amblyopy remain, stimulating lotions and vapours may produce its removal.

*Amaurosis.* Here we shall confine our attention to the disease's general character, for the differential traits which mark its varieties, we refer "surgeons commencing practice" to Weller's "Manual," or the more accessible repositories of rudimental pathology.

Amaurosis, then, is either partial or complete: it exhibits a twofold set of symptoms—the *subjective*, those perceived by the patient himself—the *objective*, such as come under the surgeon's observation.

1st. In the subjective, vision in one or both eyes declines, or is lost; dryness of the organ, sense of dust under the eye-lids, and a feeling as if the bulb were forced out of the socket, are experienced; painless irritation, fulness and weight in the ball and its appendages, with head-ache and giddiness, ending in decrease of sight, are present; these sensations frequently precede, or are co-existent, with incipient blindness; when pain is extreme, inconsciousness and delirium are concomitant; and in such cases extensive organic lesions of the bones or brain, particularly of the cranial bones, are discovered; when memory and perception begin to fail, death soon ensues; vision, moreover, may be hemioptic;* ateloptic, myodesoptic, photoptic, photophobic, oxyopic, nephodic, diplopic, chruptic, strabismal, or luscitant; sometimes patients are short-sighted, sometimes far-sighted, at other times see objects mis-shapen or displaced.

2d. In the objective, the pupil is often too large, occasionally too small, almost always distorted and angular; at one time, it is smoaky and clouded, at another dark-grey or greenish-grey, and not seldom reddish or yellowish-white, when it obtains the cataractal opacity; in all these cases, the muddiness appears distinct in the posterior part of the eye and visibly concave; generally the iris is immoveable; when one eye only is amaurotic, both pupils sometimes contract and expand so long as both eyes are open; but if the second be shut, the other's iris is immediately fixed, and the pupil angular and enlarged; lethargy often, and insomnolency, with paralytic and convulsive symptoms, supervene; and when the latter arise, an unfavourable issue impends.

Manifold and various are the causes of amaurotic blindness, but on their particularization we decline to dwell. Beer, and after him Weller, enumerates the varieties of Amaurosis under four generic and eighteen specific distinctions. 1. Dynamic amaurosis, whose symptoms are subjective only, and the eye remains unaltered both in structure and form; this arises from over-excited vitality of the ocular nervous textures, and from the vitality of the optic nerve being too much depressed. 2. Amaurosis, wherein the ophthalmic organization is morbid—this is the aluroptic (cat's-eye) amaurosis which has been theoretically regarded as dependent on absence or defect of the choroideal paint. 3. Amaurosis from malformation of the eye in general, or of its individual parts, especially of its irritable tissues;—it is excited by immoderate use of bitter or narcotic food or medicine, and the improper application of lead; it is symptomatic of hypochondriacal, hysterical, epileptic, convulsive, and nervous affections in all their shapes; it arises from a loaded state of the bowels; it is vicarious of acute cutaneous eruptions; it is rheumatismal; it alternates with repelled cold in the head, without a sensible collection of mucus in the frontal sinuses; it is purely paralytic; and it constitutes a symptom of hydrencephalic effusions.

---

* We have not been betrayed into the use of these and other appellatives by the unworthy influence of affectation or pedantry: our paramount object at all times is, to diffuse the greatest proportion of useful knowledge in the fewest words and in the smallest possible space. Such motives, we know, will be rightly appreciated by the intelligent class of our profession; to the approbation or censure of others we are indifferent.

4. Mixed amaurosis, in which the characters of the former three kinds are blended;—this is traumatic, proceeding from injuries of the ocular region; it is arthritic, being determined by neglect of the body and mind, in a gouty habit; it takes place after quickly repressed and cured eruptions on the head, old ulcers of the feet, in itch, and in plica polonica, when the hair has been shaved; it is an immediate consequence of a very severe and suppressed fit of passion; it follows repressure of encyphalic catarrh with deposition and matter in the sinuses of the frontal region; sometimes, though rarely, it is sequent to an abrupt interception of the lacteous secretion, during the puerperal state, and it originates from structural lesion of the optic nerve and of the skull and brain. Each of these amaurotic conditions is particularized by a characteristic definition; its etiological and prognostic distinctions are superadded, and its management illustruted by a circumstantial series of hygienic and therapeutic rules. However valuable and instructive these precepts may be, it is obvious that we cannot here undertake their transcription; we shall therefore dismiss the subject with a concise exhibition of the treatment wherewith Dr. Weller proposes the disease's general phenomena should be mitigated or repressed.

When the causes* of Amaurosis have been discovered and their nature and degree duly considered, their removal must be attempted by suitable means. If, however, it is impossible to ascertain them, then says Dr. Weller, nothing remains for our guidance but well-regulated empiricism, in which the age and sex and the diseased appearances with which the amaurosis originated, are to be taken into consideration. By this rule, then, emetics may be employed to remove gastric impurities, or to operate a change upon the nerves;—purgatives, to regulate the assimilative functions, and to relieve sanguineous congestion in the head;—diaphoretics, to open the capillary vessels, and balance inordinate cutaneous action;—emmenagogues, to induce suspended menstruation; anthelmintics, to remove the irritation of worms;—mercurials, to expel the germs of a syphilitic taint;—antiscorbutics, when the patient is of a scorbutic habit;—antineurotics, if the disease be really nervous;—and local remedies, such as abstraction of blood, issues, setons, sinapisms, blisters, electricity, galvanism, lotions, frictions, with stimulating ointments or liniments, and bathing, warm, tepid, and cold, in all its forms.

*Anomalies of sight.* These are squinting, luscitancy, or oblique vision; myopy, or short-sightedness; diplopy, or double vision; hemeralopy, or nocturnal cecity; and myctalopy, or day-blindness. When symptomatic, these states will depart with the madadies wherewith they co-exist; when otherwise, they may be expected to yield to the common mechanical agents which ingenuity and experience have supplied.

---

* Dr. Monteath has seen a variety of cases of amaurosis, both complete and incomplete, succeeding the late typhus fever; and he regards them as being exceedingly difficult of a perfect cure.

*Fungus Hæmatodes Retinæ* consists in fungous degeneration of the retina, and observes the following course:—an amaurotic amblyopy, with complete dilatation and immobility of the pupil, and aversion from light, proceeds to perfect blindness; at the same time is seen in the retinal region, a body resembling a concave silver plate, or piece of polished steel; by and by, this metallic appearance changes into a yellowish or greenish irregular speck, not unlike coagulable lymph effused into the bottom of the eye, and counterfeiting a partial opacity of the vitreous humour; the conjunctival vessels are loaded, and the eye in constant pain; furnished with blood-vessels from the central ophthalmic artery, the little greenish or yellowish mass increases by degrees, extends toward the iris, and is recognized as a yellowish, spongy substance; pain in the forehead and nape become aggravated and experiences nocturnal exacerbations; the fungous mass enters the anterior chamber, which now appears turbid; the eye-ball begins to be nodulated, and the sclerotica assumes a dark-blue or livid colour; occasionally the eye-ball about this period becomes dropsical; the growth now reaches the cornea, which is turbid and thinner from the pressure, begins to suppurate, and thus allows the fungus to project externally, attended with agonizing pain; forthwith the morbid mass acquires a reddish colour, mingled with yellow or black spots, but always retains its peculiar soft, spongy, cerebriform consistence; on the slightest touch, it throws out much blood and an offensive ichor, resembling the washings of flesh; the fungus being fairly formed, symptoms of the absorption of ichor arise; the lymphatic glands, in the parotideal, submaxillary, and cervical regions, swell; and, exhausted by an irremediable and unmitigable disease, the patient languishes, emaciates, and expires. Remedial and palliative applications are here useless; extirpation of the eye-ball is almost always unavailing; for in a short time, the malignant excrescence repullulates, or penetrates the cranium, destroys the optic nerve and contiguous tissues of the brain.

*Ophthalmitis Externa* originates from extraneous bodies entering the eye and eye-lid, superficial wounds of the eye, sting of a wasp or bee, and similar causes. It commences with diffusive conjunctival and sclerotic vascularity; painful motion and dryness of the organ and its coverings; progressive corneal suffusion and opaqueness; invisibility of the pupil and iris from turbidity and redness of the intervenient membranes; decreasing vision, actions of the eye-ball over-forward; inflammatory fever; the conjunctiva now swells and becomes dark red; the cornea acquires a white, then a yellowish colour, and finally suppurates, leaving the eye-ball immoveable, whitish, shrivelled, indented. Remove the cause if known; and, for general treatment, institute spare regimen and depletion; for topical, employ leechings, scarifications, lotions, liniments, unguents.

*Ophthalmitis Interna.* It is rare; its causes are ill defined. An oppressive tension, obtuse pain of the whole eye, rapidly acquiring intensity, and spreading over the eye-brows and head, usher in its first stage; there are flashes of fire before the eyes, decrease of vision, muddines and closure of the pupil; if grey or blue, the iris changes to green, and to red when it is brown or black; it also swells, presses towards the cornea, and lessens the anterior chamber; as the iris swells the sclerotica and conjunctiva redden; there is severe head-ache; the cornea loses its brilliancy; synochal fever prevails; by and by, suppuration begins, advances, maturates; pus is deposited in the anterior chamber; and, if this is neglected, the cornea bulges, becomes conical, and bursts at last under unsufferable pains, which subside not till the eye-ball retire. Treat this as a general ophthalmic inflammation; when pus is formed, promote its abruption; place blisters behind the ear and on the temple; moisten the eye often with vinous laudanum; open the cornea with a lancet only when disruption impends; and, with incarnative dressings, guide the ulcer to granulation and health.

*Staphyloma Corneæ* takes place, when the cornea loses its transparency, and swells; when the iris is tumid and projects; when these inflamed membranes cohere; and when secretion of the aqueous humour is not destroyed: it is total, when adhesion of the iris with the cornea is general; partial, when a portion only of these tunics is united: in the latter, vision sometimes remains; in the former, blindness is certain and life insecure. Astringents, caustics, pyrotics, and such means offer but a doubtful obstruction to the development of a staphylomatous tumour; even the operation seldom succeeds. When this is indicated, Beer's method may be preferred. With a cataract-knife, he perforates the tumour's base from the temporal to the nasal angle, and completes the flap by pushing on the knife horizontally, as in extraction. During this process, an assistant holds up the superior eye-lid, and must not allow it to fall down, after completion of the first cut. The operator now seizes the flap with strong forceps, and cuts it away with scissars, avoiding pressure on the eye as the occasion of losing the lens and vitreous humour. The eye-lids are now to be closed, and simple dressings, as in cataract, applied. In operating for conical staphyloma, the lens and vitreous humour can never be saved, because the incision is made behind the lens; the tumour also, in this case, should be hooked at the commencement, and excided in the way already described.

*Staphyloma Iridis.* Projections of the iris have different names according to their bulk;—they are *myocephalous*, having the size and shape of a fly's head;—*hylomic*, when flattened, slightly elevated above the cornea, and resemble the head of a nail;—and *racematous*, when the iris penetrates through several openings in the cornea. Its management varies little from that of the preceding; only when the tumour has a pedicle or is insensible and callous, or the eye varicose, escharotics must give place to the knife.

*Synechia.* This is always a result of ophthalmic inflammation. It is either—*anterior*, when the iris comes in contact with the cornea and undergoes adhesion—or *posterior*, if the capsule of the lens is attached to the iridial surface. The former, when ancient, is incurable; if vision is not much deteriorated interference is improper; for its removal, perforate the cornea with a cataract-knife, separate the adhesion from below upwards, preventing, at the same time, premature escape of the aqueous humour. When *total*, the latter is irremediable and combined with cataract; when *partial*, it is sometimes cured by external and internal medicines.

*Ophthalmitis Generalis.* Its symptoms are severe, pressing, tensive pain in the eye and its appendages; increasing conjunctival and sclerotic redness; conjunctiva of the eye-ball swells and forms an equable, irritable, hard wall around the cornea, whose centre at last can hardly be discerned; the pupil is contracted, the iris immoveable, vision nearly or altogether lost; photopsy augments the local pain; the blue or grey iris becomes greenish, and the brown or black changes to red; the eye-ball enlarges and loses its mobility; the cornea grows muddy, then opaque; the eye-lids inflame, the under one is everted; secretion of tears and mucus ceases; inflammatory fever, with suspension of consciousness, predominates. The second stage now sets in, with augmented conjunctival tumefaction; it grows dark-red, but softer; the pain is irregular, pulsating, pricking; the upper eye-lid is stretched, and acquires a bluish-red colour; the visible part of the cornea becomes snow-white, then yellowish; weight and coldness are felt in the eye and orbit; the eye-ball, if unassisted, bursts with an audible report, and discharges the remains of the lens and vitreous humour; and in some instances the scene is closed by the supervention of gangrene, sphacelus, and death.

Venesection, alvine evacuations, topical leechings, and refrigerant lotions, radiated scarifications of the conjunctiva, and, if there be encephalic excitement, puncture of the jugular vein, will, if it is attainable, promote resolution. When suppuration is imminent, lenitive poultices and internal tonics will accelerate its course. Gangrene must be opposed by cataplasms formed of warm, concentrated decoction of bark, treacle, Peruvian balsam, and vinous laudanum, with the internal use of cinchonal, ethereal, and similar medicines.

*Atrophia Bulbi.* It will not be mistaken; very often it is a consequence of arthritic ophthalmitis; it is incurable: when the atrophic eye-ball can bear the irritation, an artificial eye may be introduced.

*Cirsophthalmia.* This state is often induced by frequent or neglected inflammations of the eye-ball; in it, although a sense of light fails, flashes of different colours are observed; the eye-ball is indurated, conical, enlarged; its lids no longer cover it; its white changes to dirty blue, or is lead-coloured; the conjunctival, sclerotic, and choroidal vessels are varicose and replete with dark blood; the cornea becomes opaque or staphylomatous; the iris is fixed; motion of the eye-ball slow, the pupil dilated and angular, with a muddiness perceptible behind it. Interfere not with these symptoms; they may exist long without an increase; strong and corrosive applications irritate the eye; its largest varicose vessels burst, and the chambers fill with extravasated blood; even rupture of the eye-ball itself, with alarming hæmorrhage, carcinomatous change of structure, misery and death not unfrequently ensue.

*Scirrhus and Cancer.* Nodulated, irregular, whitish-red, indurated unpainful tumefaction of the eye-ball, announces the scirrhous condition; the ophthalmic vessels are varicose, turgid with violet blood; as cancer is established, the ocular tunics become thickened and sarcoidal; a stinging, itching, burning extends all over the head; ultimately, the eye-ball changes into an irrecognizable mass, resembling hard flesh; its size enlarges; hectic fever is kindled; one or more cancerous ulcers form; excruciating pain is experienced; hæmorrhage from ruptured varicose vessels is frequent and exhaustive; the ulcers acquire a wart-like base, with everted, hard, lead-like edges, and discharge a nauseabund ichor, which is the certain forerunner of dissolution. Alleviate the predominant symptoms, while palliation avails: as the last, but equivocal recourse, extirpation of the eye may be performed.

*Ophthalmic Ossification.* The sclerotic and choroideal coats of the eye have been found in an ossified state, with complication; the iris also, the retina, the optic nerve, and even the whole eye-ball, have been converted into bony structure.

*Congenital Deficiency or Superfluity of Eyes* not unfrequently occurs. Dr. Monteath, at vol. ii, p. 207, in a foot-note, relates a remarkable instance wherein three out of eight children were born without any vestige of eye-balls. Their mother, previously to her marriage, was some time servant in the family of the writer of this article, who attended the daughter during her last illness. Both parents are perfectly formed, healthy, and virtuous.

*Ophthalmia Catarrhalis.* The eye-lids in this redden, and experience a burning sensation which occasionally is diffusive; the eyes shun the light, and swim in acrid tears; sandy particles seem inserted between the eye-ball and lids; on these symptoms decreasing, the meibomian glands throw out bland muco-puriform matter; sometimes there is spasmodic winking, with implication of the lachrymal sac; the disease moreover increases in the evening, and is thus distinguished from scrofulous inflammation of the eye. Allay the local symptoms with frigid cataplasms, and leeching of the nasal angle; or, after our British manner, bleed from a brachial or jugular vein, till fainting is induced. For checking the inordinate glandular secretion, diaphoretics may be given internally, with topical applications of styptics, aromatic herbs, and astringent salves.

*Ophthalmia Rheumatica.* Its opening phenomena resemble those of the preceding kind; heat aggravates the pain; the vascularity and lachrymation augment; watery vesicles form in the corneal or albugineal membrane; the former becomes dull and muddy; in the end, these vesicles burst, and emit a thin acrimonious discharge, from ulcerations, various in their inveteracy and extent. Meet it in the first stage with topical depletion and resolvents, and with mild diaphoretic remedies, internally exhibited; when stronger means are required, guaiac, camphor, leopard's-bane, and antimonials, with cervical and post-auricular vesications, and opiate plasters, may be tried; the ichorous ulcers, if the general health is maintained, will yield to the usual applications.

*Ophthalmia Arthritica* assumes an erysipelatous, an iritic, or sclerotic character. In the former, on repression of gout, the eye-lids experience burning pain, and a pale-red vesicular swelling, which soon arises into a bladder containing yellow acrid lymph; this tumefaction extends to the conjunctiva of the eye-ball and lachrymal sac; acrid tears now begin to flow, but are soon displaced by a blepharo-blennorrhœal and ophthalmo-blennorrhœal discharge, which proceeds rapidly and unremittingly to destroy the eye by colliquation. Recal the suppressed gout with pediluvia and sinapisms round the feet, vary the common topical applications according to circumstances, and let a diaphoresis be promoted. Its two latter forms carry the marks of sclerotic and iridial inflammation, but in an aggravated degree. They are to be managed as such, and particular attention, at the same time, paid to the primary disease.

*Ophthalmia Variolosa* is—1st. Variolous blepharophthalmy, wherein, as the small-pox appears, the eye-lids swell, close, and are covered with pustules, which dry about the ninth day, and the eyes re-open; in some patients, the conjunctiva inflames; there is photophoby and dryness, with sense of foreign bodies in the eye. Here defend the eye from irritating agents; promote derivation by blisters, open the pustules when maturated, and wash them with tepid milk or mucilaginous opiated eye-water, exposing the eye at the same time to dry warm air and moderate light. 2d. External variolous ophthalmy presents itself with conjunctival and sclerotic redness, great aversion from light, stinging pain, and a flow of tears; turbid points appear in the cornea, and the iris is affected; on the vascularity and retinal irritableness subsiding, the cornea loses is transparency, and genuine pustules form on its opaque parts. Prevention of this disease is strongly indicated; and, for this purpose, camphorated compresses should be placed over the eyes, which are to be frequently moistened with lard or solutions of the acetite of lead; the inflammation may be treated with cold aqueous poultices, and a blister or cautery behind the ears; the pustules require being opened, the ulcers assisted to granulate and heal; and the powers of restoration supported by exhibitions of aromatic, ethereal, cinchonal, and opiaceous drugs. 3d. Glandular variolous ophthalmy usually begins with a blepharo-blennorrhœal, and terminates in an ophthalmo-blennorrhœal state, which is to be removed by modifications of a suitable treatment.

*Ophthalmia Morbillosa et Scarlatinosa.* Little dissimilarity is perceivable in the symptoms of these inflammations. They commence with conjunctival and sclerotic erubescency, photophoby, effusion of acrid tears, deep stinging pain on the eye, glittering spots on the cornea, and secretion of pungent nasal mucus; with aggravation of these, the palpebral margins become red and irritable; conical watery vesicles arise on the cornea; these burst and form ulcers, from which issues a thin acrimonious discharge. Keep the eye uninfluenced by every stimulating cause; and, to avert the formation of ulcers, place blisters behind the ears or on the arms; when the vesicles burst, and the ulcerative process is declared, appropriate lotions and salves, with constitutional remedies, will come to be required.

*Ophthalmia Syphilitica.* Sometimes it is a sequence of confirmed lues, sometimes an irregular gonorrhœal symptom. In the first, it appears as a syphilitic iritis or a syphilitico-scorbutico-blennorrhœal ophthalmy; in the second, it always shews itself as a blennorrhœa of the eye-lids and eye-ball.

*Ophthalmia Gonorrhoica.* It is either vicarious of a venereal clap, or proceeds from the eye being infected with gonorrhœal matter. It begins, at various intervals after suppression of the urethral discharge, with a bright red, hard, painful swelling on the edges of both eye-lids, which is forthwith propagated to the conjunctival and sclerotic textures, and throws a hardish, tile coloured zone around the cornea; intolerance of light and the ophthalmic pain increase; head-ache and feverishness predominate; at an uncertain stage of the disease, the palpebral and ocular conjunctiva pours out a whitish, then a yellowish or greenish-yellow mucus in profusion; swelling of the upper eye-lid increases in a frightful manner, becomes livid, and hangs over the eye-ball like a lump of flesh; at last, matter forms in the eye, the corneal lamellæ give way and open like the leaves of a much-read book, the cornea itself bursts, and complete colliquation of the eye ensues. At the commencement, if the disease is vicarious of clap, let a general and topical antiphlogistic treatment be instituted, and the gonorrhœal discharge established. For this purpose, every two hours, apply poultices of bread, milk, saffron, and henbane, to the penis, and frequently bathe it with tepid water; practise dry cupping on the perinæum; inject lukewarm oil into the urethra, and lodge in it bougies either dry, or smeared with salve of muriated quicksilver, or gonorrhœal matter.

*Syphilitic Iritis.* Persons who suffer from confirmed lues, or in whom former luitic symptoms have ceased, become afflicted with this disease; it seldom implicates the internal ocular tissues. Its symptoms are a pale sclerotic redness, forming a broadish zone around the corneal verge; dimness of the cornea, immobility of the iris, and angular contraction of the pupil; the iris is swollen, reverted, projected; there are aversion from light and lachrymation; osteocopical pains, which commence at sun-set, become tense at midnight, and decline at five in the morning, spread from the nasal along the supraorbitary arch, to the temporal angle; shreds of coagulable lymph appear behind the pupil, and gradually extinguish sight; condylomatous swellings arise on the ciliary and pupilary edge of the iris; and, at the same time, fatty ulcers form upon the cornea and white of the eye. The cure consists in removing the nocturnal pains of the bones by friction of the superciliary regions with opiated mercurial ointment, and placing on the eye a warm linen compress; at the same time, the nitric oxide or submuriate of quicksilver with opium, may be exhibited internally, care being taken to avoid salivation and purging, if there is apprehension for the patient's lungs; and ichorous ulcers require being treated with tepid astringent lotions or the red mercurial salve.

*Ophthalmoblennorrhœa Syphilo-Scorbutica.* Neglected syphilis, debauchery, and filthiness, producing scorbutico-cachectic debility, occasion this disgusting complaint. Its pathetic accompaniments are—an immense bluish-red irritable, inflammatory swelling of the palpebral edges, without a previous or suppressed gonorrhœal discharge; troublesome itching and burning of the eye-lid; the conjunctiva perfectly violet, and swelling like a vesicle into unequal prominences around the cornea; immobility of the eye-lids; the lower forms a violet coloured, spongy ectropium, which readily bleeds; excessive flow of muculent fluid from the upper; a weak, intermitting pulse, bleeding gums, fetid breath, black corroded teeth, and complete blindness from disorganization of the ophthalmic parts. Here the treatment is the same as in pure blennorrhœal ophthalmy; only, mercury is distinctly contra-indicated, because in the smallest dose it excites injurious salivation, or colliquative diarrhœa, which often endangers life.

*Psorophthalmia* originates from repression of the psoric eruption, or from infection of the eye-lids with the matter of itch. It is distinguished by a dark-red swelling on the palpebral edges, severe itching, and numerous pustules bestudding its surfaces; these burst and become ichorous ulcers, accompanied with hot itching pain; subsequently they enlarge under the superincumbent crusts; fresh ones successively appear, and overspread the tumefied eye-lid, giving it a dasymatous appearance; the ulcers penetrate deep into the palpebral margins; the ciliary bulbs are lost; and trichiasis or an incurable eutropium remains. Lotions of warm infusion of water-germander, and inunction of the parts with sulphureous, camphorated, and mercurial salves, may form the basis of the local treatment; the general will consist of the internal exhibition of antimony, sulphur, camphor, together with sulphureous baths and frictions of the body, the parts near the eye or those behind the ear, with an antimonial unguent; when the psorophthalmy resists all curative measures, the original cutaneous disease must be reproduced.

*Ophthalmia Scrophulosa* occupies the eye-lids, or anterior part of the eye-ball, or the lachrymal sac, or is complicated. An unusual secretion of viscid mucus from the meibomian glands, slight redness and tumefaction of the eye-lids, lachrymation, a sense of burning, and aversion from light, are among its first symptoms; the tears become acrid and excoriate the surrounding parts, which experience an œdematous distention; the conjunctiva now inflames, and is covered with diffused redness; clusters of blood-vessels run concentrically towards the corneal centre, the sclerotica reddens, and pustules appear at the extremity of the vascular clusters, and sometimes change to ulcerous or fungous sores; then ulcers perforate the cornea; the iris falls into the opening, and a staphyloma is produced; sometimes watery vesicles arise, burst, and give place to a corneal hernia; there are painful spasms of the eye-lids; and, not unfrequently, iritis or total staphyloma of the cornea supervenes. Whoever possesses a panacea for scrofulous disease will here bring it into requisition, as the general treatment of this ophthalmy. Our practice boasts but a precarious efficacy, and we have less expectation of advantage from that which Dr. Weller recommends. As topical remedies, warm poultices, fomentations of hemlock, poppy, or henbane, narcotico-mercurial salves, mucilaginous and slightly astringent eye-washes, with leeches and scarifications, may be opposed to the various phenomena as they obtain.

*Dacryoblennorrhœa.* When it exhibits manifest symptoms, there are —dryness of the nostril; inflammation of the lachrymal sac; a hard bean-shaped swelling in its site; and this, on being formed, discharges a mucous fluid through the lachrymal points. When it has frequently relapsed or become habitual, it is irremediable; sometimes, it experiences a spontaneous cure; occasionally, it terminates in obliteration, fistula, hernia, or dropsy of the lachrymal sac; and each of these conditions solicit being removed ere its development is complete.

*Ophthalmia Scorbutica.* Aversion from light and dislike to glittering objects prevail in this disease; the sclerotica is bright-red, the conjunctiva vascular and varicose; the cornea and aqueous humour turbid and cadaverous; the iris projects, its vessels are morbidly dilated and trace a concentric course; without being much expanded or contracted, it becomes immoveable; the eye moves with difficulty; conjunctival extravasations of blood spontaneously form; blood appears in the anterior chamber; vision, hitherto weak, now disappears; sclerotic staphylomata in the form of dark-blue swellings, rise around the cornea; epistaxis supervenes, together with a flow of tears resembling the washings of flesh; and all the other symptoms of scurvy follow in its train. Perfect ophthalmy, in a scorbutic constitution, is incurable. Here the eye does not bear local medicaments; a general tonic treatment, however, may be instituted with the object of renovating the patient's powers, and thus gradually exterminating the disease.

Thus we have endeavoured to place before the British profession a comprehensive and impartial view of Dr. Weller's ophthalmographical pathology. Conceiving it to be made unnecessary by the space we allotted, in the two last numbers of this journal, to an exhibition of our native practice, we have taken occasion, in few instances throughout this long article, to advance comparative opinions. To our readers, therefore—before whom we have adduced ample evidence—we resign the task of estimating the merits and defects of both systems: let them profit, by what is useful in either, and let them improve themselves by what is new.

At parting with the translator's elegant and expensive volumes we find pleasure in offering our admiration of the correctness and beauty of their typographical execution. Mr. Chapman has long and justly flourished as the BENSLEY of Strath-Clyde, and we shall not endanger our reputation for bibliosophy and taste, if we pronounce this work of his to be unsurpassed by the best specimens of professional printing which have ever issued from any provincial or metropolitan press.

*Note.*—P. 640, line 3 from bottom, for *Stannus* read *Pannus.*

